# Assessing bias in measuring power outage exposure with simulations

**DOI:** 10.1097/EE9.0000000000000403

**Published:** 2025-06-11

**Authors:** Heather McBrien, Daniel Mork, Marianthi-Anna Kioumourtzoglou, Joan A. Casey

**Affiliations:** a Department of Environmental Health Sciences, Columbia Mailman School of Public Health, New York, New York; b Department of Biostatistics, Harvard T. H. Chan School of Public Health, Boston, Massachusetts; c Department of Environmental and Occupational Health Sciences, University of Washington, Seattle, Washington; d Department of Epidemiology, University of Washington, Seattle, Washington

**Keywords:** Power outages, Simulation, exposure assessment, Bias, Electrical customers, Poweroutages.us

## Abstract

**Background:**

New national power outage exposure data have become available since 2020, which can support epidemiologic studies of power outage and health outcomes, but exposure assessment challenges remain. Two sources of bias could affect results: available datasets are missing large percentages of observations, and the health-relevant duration of power outages remains unknown. Here, we aimed to determine if existing datasets can produce usable effect estimates in epidemiologic studies despite missing data, and quantify bias introduced by incorrect assumptions about the health-relevant duration of power outages.

**Methods:**

Based on existing data from PowerOutage.us, we conducted simulations representing a county-level study. We simulated and then estimated the effect of daily power outage exposure on hospitalization rates. We measured the magnitude and direction of bias introduced in the presence of incorrect assumptions about the health-relevant power outage duration and when increasing amounts of exposure data were missing.

**Results:**

When the health-relevant power outage duration was underestimated, results were substantially biased towards the null (mean bias: −64.7%, SD: 34.9). Overestimation of the health-relevant power outage duration resulted in smaller bias (mean bias: −6.7%, SD: 30.6). When 80% or more of county-level person-time of power outage data were missing in 80% of study counties, results were severely biased towards the null (mean bias: −54.4%, SD: 39.8).

**Conclusions:**

Our results show that while some bias is likely, sensitivity analyses and careful choices of health-relevant duration can help researchers leverage available power outage data to produce low bias effect estimates in epidemiologic studies of power outages and health outcomes.

**What this study adds:**

Research on risks of power outages to health has been constrained by a lack of exposure data. Since 2020, new national power outage exposure data have become available. However, these new datasets are missing large amounts of data. We conducted a simulation study assessing how missing data could bias results of an epidemiologic study of power outage and health, testing if the new power outage data were usable. We found missing data can introduce substantial bias, but it is possible to estimate effects with low bias from available datasets by restricting use to locations missing less person-time outage data.

## Introduction

Power outages are becoming more common.^1,2^ Climate change has increased the frequency and intensity of extreme weather, such as heat, wind, and precipitation.^3,4^ Aging electrical grid components have not been modernized to withstand these previously rare severe weather events.^2,5,6^ As a result, US electrical customers experienced an average of 8 hours without power in 2020, the longest duration on record.^6^

Power outages can pose serious acute health risks to vulnerable people. For those who use life-sustaining electricity-dependent medical equipment such as at-home ventilators and oxygen tanks, loss of electricity can be life-threatening.^7^ In children, outages can increase accidents and injuries related to generator, natural gas, and candle use.^8–10^ Power outages can render air conditioners, heaters, and tap water unavailable, resulting in heat exposure, cold exposure, and dehydration. Older adults are especially susceptible to stroke, myocardial infarction, chronic obstructive pulmonary disease exacerbation, and other adverse cardiorespiratory outcomes following such exposures.^11–15^

Despite the health risks of power outages, data describing power outage exposure are limited,^2,16^ constraining research. To our knowledge, only one US-based dataset describing outage exposure has been used to evaluate the impact of power outages on health.^8,14,15,17–21^ This dataset describes outage exposure across space and time at a subcounty spatial scale in New York State.^8^ Almost all published studies of power outages to date rely on this single dataset,^2^ meaning results are specific to New York State and may not be generalizable. The remaining studies of outage and health use large-scale events such as single hurricanes or other disasters that disrupted power as a surrogate for the timing of power outage exposure in specific locations.^18,21^ These studies consider everyone in a city or county exposed to the power outage in the hours, days, or weeks following the index event. Unfortunately, studies based on single climate events cannot disentangle the health effects of power outage exposure from simultaneous severe weather exposure, and they cannot be used to estimate exposure-response relationships between the severity of power outages and health. This would require measuring power outage exposure both in space and time.

Quantifying the health risks and costs associated with power outages can influence energy policy decisions. If power outages cause significant morbidity and mortality, improving grid infrastructure, community solar power, electricity storage, and electricity reliability could cost-effectively improve community health. In addition, power outages may mediate the effects of climate hazards such as heat waves and storms on health, and improving electricity reliability during these events could lessen their health impacts and improve climate resilience. Knowing the health risks of power outages can also motivate interventions in vulnerable populations such as older adults and children to prevent adverse health outcomes.^7^

In our previous work, we created a new national dataset of hourly power outage exposure by county in the continental United States^22^ (the PowerOutage.us dataset or POUS dataset). This dataset could allow researchers to characterize exposure-response relationships between power outages and health outcomes nationally, by region, and within vulnerable populations.

However, even with these new data, major challenges with exposure assessment remain. First, there is no established strategy to measure health-relevant power outage exposure in the literature.^2,16^ Power outages are not spatially continuous exposures, such as air pollution or heat. Individual households or grid connections in the same area may not experience power outages at the same time. However, the only data currently available to assess power outage exposure are counts of customers without power by hour at an aggregated spatial unit level (e.g., county), which can be interpreted in multiple ways. When assessing spatial unit-level exposure, researchers must select a cut point (a percent of customers in a community without power) after which an area is considered exposed to a power outage. They may also consider and define the relevant power outage duration (i.e., how long does an outage need to last for a spatial unit to be considered exposed?). A single strategy to describe power outage exposure would allow comparability and pooling of results across studies.

Second, the health-relevant power outage duration matters for exposure assessment. Many existing studies examine outages of a specific length (e.g., 8+ hours).^8,16,20^ However, we are unaware of literature or other knowledge describing how long power outages must last to cause any adverse health outcome. There are likely threshold effects: power outages longer than a certain duration may increase the risk of an adverse health outcome, but shorter outages may not. For example, 8+ hour power outages may affect the health of those using oxygen tanks and at-home ventilators if device batteries die after 8 hours of power outage, while shorter outages may have no effect. The health-relevant duration is likely different for different health outcomes. Incorrect assumptions about the health-relevant duration can potentially bias the results of epidemiologic studies of power outages and health outcomes.

Finally, both the New York State (NYS) and POUS datasets are missing large percentages of observations,^8,16^ with some counties in POUS missing data on up to 70% of county-hours. In the POUS dataset, data are missing when utilities did not have a website or if utility websites were offline for long periods of time. In these cases, imputing missing values is nearly impossible because no data exist from which to draw information. Researchers face decisions about how and when to exclude areas with poor coverage while balancing generalizability and aiming to minimizing bias.

In this paper, we aimed to address these exposure assessment issues. First, we developed a strategy for assigning power outage exposure. Then, we ran simulations to address two potential sources of bias: incorrect assumptions about the health-relevant outage duration and potential bias due to missing data. We quantified the direction and magnitude of bias introduced when a certain length of power outage is assumed to cause adverse health outcomes (e.g., 8+ hours), but the true health-relevant duration was different (e.g., 4+ hours). To deal with missing data, we used simulations to identify patterns of missing data that would bias outage-health effect estimates. We tested the sensitivity of simulation results to effect size and study design, using effect sizes estimated by previous studies of power outage and health outcomes and study designs that researchers could use to conduct epidemiologic studies of power outage and health outcomes.

Our results contribute to the power outage and health literature with recommendations for consistently defining and measuring power outage exposure, using the datasets currently available while minimizing potential bias in future epidemiologic studies. Our results also inform the interpretation of previous studies conducted with these existing power outage exposure datasets.

## Methods

### Power outage datasets

In our previous work, we purchased raw power outage data from PowerOutage.us to create a new national dataset of power outage exposure^16^ (the POUS dataset). Most utility websites report the number of customers without power by neighborhood or city in real time. PowerOutage.us compiled these data by scraping counts of customers without power from utility website application programming interfaces covering the US states in real time every hour from 2018 to 2020.^22^ We used this compilation to produce the POUS dataset, containing hourly counts of customers without power for US counties (n = 2447 [78%]) from 1 January 2018 to 31 December 2020.^16^

Utilities define a “customer” as a grid connection, which can correspond to a household, apartment building, or business—one customer can represent multiple individual people.^22,23^ Counts of customers without power (henceforth, “customers out”) reported in this dataset do not necessarily track the same customers: if 10 customers are reported out in two subsequent hours in one county, the data do not contain information about whether the same 10 customers lacked power. For example, 10 customers may have been without power for 2 hours, or 10 customers may have been without power in the first hour and a different 10 customers were without power in the second hour, meaning 20 customers were without power for 1 hour each.

The NYS power outage dataset is structured similarly—counts of customers without power are reported every 30 minutes by power operating division.^17,19^ Power operating divisions (n = 1865) are geographic units varying in size but similar to ZIP codes throughout the state.

### Strategy to assess power outage exposure

Data from POUS contain counts of customers without power by hour within each county. When using POUS to study power outage exposure and health outcomes, a naïve approach would be to estimate the association between total daily customer-hours without power and daily county-level health outcomes. However, “customer-hours without power” is not a well-defined assessment of exposure. For example, if one county-day has 1000 customer-hours without power, this could mean that 100 unique customers were without power for 10 hours total during the day, or 1000 unique customers were without power for 1 hour. These two interpretations of “customer-hours without power” would likely have different implications for health.

Because customer-hours without power is not well defined, it would be difficult to interpret the meaning of effect estimates from a study using this exposure metric or shape policy based on this exposure metric. In our proposed strategy to assess exposure to power outage, we aimed to summarize continuous counts of customers without power so that we captured both dimensions of area-level power outage exposure: the magnitude of outage (how many customers are affected) and the duration (for how many hours in a day).

**Figure 1.**
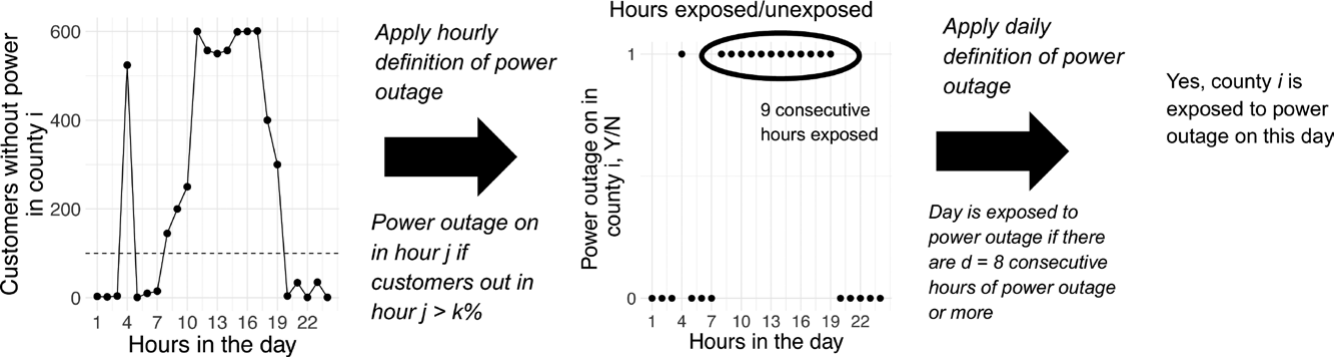
Example of data summary process to identify if a county-day were exposed to a power outage. In this example, county *i* has 1000 customers. The leftmost plot shows counts of customers without power in county *i* for each hour of the day in question. The dashed line on the leftmost plot represents *k*, the cut point after which an hour is considered exposed to power outage (10% in this case, or 100 customers). The center plot shows hours exposed/unexposed to power outage, with 9 consecutive hours of the 24 hours exposed. Because this is longer than 8 hours, the specified duration, this day would be considered exposed to power outage.

To determine if a county-day was exposed to a power outage (Figure 1), we first considered each hour alone. We considered a county-hour exposed if the percentage of customers without power in county *i* during hour *j* exceeded an arbitrary cut point *k%*—for example, 10% of county customers. In this example, we would define a county *i* exposed to a power outage during hour *j* if more than 10% of customers served in county *i* were without power, thus capturing outage magnitude. Then, we assumed a health-relevant duration *d* (for example, *d* = 8 hours). *d* could be any duration specified by the researcher. Then, we summarized to the daily level and considered a county-day as exposed if there were at least 8 consecutive hours of ‘power outage on’ (>*k*% customers out in county *i*) in that county on that day or ending on that day.

When a county is exposed to an 8+ hour power outage according to this definition, it does not necessarily mean that 10% of individual people in that county were without power for at least 8 hours that day. Unique customers are not tracked over time. Different customers may comprise the 10% of customers without power in different hours of the 8+ hour duration. In addition, customers may represent more than one person (e.g., if a customer is a household). Therefore, an 8+ hour outage affecting 10% of customers indicates that for at least 8 hours that day, some 10% of county customers were without power. Therefore, this is an aggregate spatial unit-level exposure definition rather than an individual-level one.

Exposure misclassification is inherent in this definition. When the county is considered exposed, some county customers will be without electricity (at least *k*%), and others will still have electricity. Other studies of power outage exposure using a similar exposure definition have handled this exposure misclassification by conducting sensitivity analyses varying the cut point *k* above which a spatial unit is considered exposed to power outage. For example, Northrop et al^8^ considered a spatial unit exposed to power outage if more than 10% of the customers served in that unit were without power and conducted two sensitivity analyses using cut points at 20% and 30%. As the cut point percentage increases, the number of customers incorrectly identified as exposed decreases, and the specificity of this definition of power outage improves.

We suggest this strategy for measuring power outage exposure to other researchers, while also recommending that researchers conduct a sensitivity analysis on the percentage out cut point. For a well-defined continuous measure of power outage exposure, researchers could also use this definition to identify the number of hours without power in some time period by spatial unit—for example, the number of hours without power in a county-day would be the number of hours with >*k*% of customers without power.

## Simulation design

### Overview

We designed a simulation representing an epidemiologic study characterizing the association between short-term power outage exposure and short-term hospitalization rates. This study is meant to mimic a study that could be conducted using the POUS data. The outcome of “hospitalizations” is intentionally vague and could be any count health outcome hypothesized to be caused or exacerbated by power outages. We simulated daily binary power outage exposure for 100 US counties for 1 year, and we created daily county-level hospitalization rates for these counties over the same period. We generated an effect estimate of power outage exposure on hospitalization from this set of 100 counties under a zero-bias scenario and then in scenarios representing incorrect assumptions about the health-relevant power outage exposure duration and including missing exposure data. We repeated each simulation scenario (containing 100 counties) 100 times, to obtain a total of 100 effect estimates of the association between power outage and hospitalizations rates per scenario. We also repeated all simulations using two different study designs to test the sensitivity of results to different model specifications, and three different effect sizes to test the sensitivity of results to effect size.

### Exposure and outcome data

For all simulation scenarios, we populated each of the 100 counties with electrical customers, drawn from the empirical distribution of customers served by county in the POUS dataset. To generate hourly counts of customers without power, we drew from the empirical distribution of counts of customers without power in the POUS dataset. Though the exposure assessment and outcome data sometimes changed throughout simulation scenarios to model incorrect assumptions about power outage duration and missing data, we used the same set of 100 counties populated with county customers (100 counties and 100 simulation repetitions) and raw counts of customers without power for each scenario.

We chose 8 hours or longer (8+ hours) as the health-relevant duration of power outage for our simulations. This was somewhat arbitrary—in a real study, the health-relevant duration would depend on the actual outcome being studied and how power outages are understood to affect that outcome. We hypothesized that 8+ hour power outages matter for electricity-dependent medical device users, so here we chose 8+ hours. We applied our definition of power outage exposure to the simulated exposure data and identified county-days exposed to 8+ hour power outage. This produced a 1-year daily time-series of binary power outage exposure data for each county.

We generated outcome data based on these exposure data. We drew hospitalization counts for each county-day based on a Poisson distribution with a base daily hospitalization rate of 0.1%. We increased this hospitalization rate for county-days exposed to 8+ hour outage by 1%, for a total hospitalization rate of 0.101%, based on reported effect sizes in the literature.^8,20^ This produced a 1-year time series of daily hospitalization rates for each county. We repeated this procedure of creating outcome data twice, with base hospitalization rates of 0.1% and rates on exposed days of 0.105% and 0.15%, to test the sensitivity of results to effect size. This produced two additional sets of outcome data for each county-year.

### Simulation study design

First, we used a base case (unbiased) scenario to estimate the true simulated effect of county-day 8+ hour power outage exposure on county-level hospitalization counts. We implemented a study design representing an augmented difference-in-differences design,^24^ where multiple counties exposed at different times are each compared with unexposed counties. Because we did not simulate any confounding, there was no need to choose counties with parallel trends during pretreatment periods, rather, we randomly chose a control county for each exposed county. For each county-day exposed to a power outage, we chose a control county-day not exposed to a power outage. We ran a Poisson model including these case and control days from all 100 counties. We repeated this modeling twice with additional outcome datasets, with base hospitalization rates of 0.1% and hospitalization rates on exposed days of 0.105% and 0.15%, to test the sensitivity of results to effect size.

We also repeated the simulation using a different study design to test if the simulation results were sensitive to study design, for all three effect sizes. We used the same exposure and outcome data generated for each of the difference-in-differences simulations but applied a different modeling procedure to the data. We used a case-crossover design with a conditional Poisson model.^25^ Within each county, we chose control days for each day with non-zero hospitalization count (i.e., each case day). We matched controls based on county and month. Since we did not simulate any seasonal or day-of-week trends in our data, we chose controls this way only to replicate how real epidemiologic study using a case-crossover conditional Poisson design might have chosen controls. We included these case and control days in a conditional Poisson model relating power outage exposure to hospitalization rates. In this model, we included an offset for customers served by county. In reality, as we describe above, in available power outage data county customers are not the same as individuals eligible to be hospitalized. However, to simplify the simulation, here we treated customers and individuals as the same.

Altogether, this produced 100 estimates of the effect of 8+ hour power outage on county hospitalization rates, for three effect sizes and two study designs (6 × 100 effect estimates in total).

### Testing incorrect assumptions about the health-relevant outage duration

To test bias introduced by incorrect assumptions about the health-relevant duration of outage, we simulated two scenarios, where the researcher overestimated and underestimated the true health-relevant duration of outage. We modeled a case where the assumed health-relevant duration of outage was 8+ hours, but the true relevant exposure was 4+ hours, and a case where the assumed duration was 8+ hours but the true relevant exposure was 12+ hours.

To do this, we created two additional power outage exposure datasets for each simulated county. Using the exposure assessment strategy above, we identified county-days exposed to 4+ hour power outages and 12+ hour outages, respectively, instead of 8+ hour outages. We then generated two additional datasets of outcome data, increasing hospitalization rates when counties were exposed to 4+ hour outages or 12+ hour outages.

Then, we mismatched the exposure and outcome data: we paired exposure data identifying 8+ hour power outages with outcome data generated based on 4+ hour outage exposure or 12+ hour outage exposure, inducing nondifferential exposure misclassification.

We then estimated the association between 8+ hour power outage exposure and outcome data generated based on 4+ hour power outage exposure or 12+ hour power outage exposure. We estimated effects using both the difference-in-differences and case-crossover designs described above, and repeated simulations for three effect sizes, where the base hospitalization rate was 0.1%, and the hospitalization rate was increased to 0.101%, 0.105%, or 0.15%.

### Testing bias due to missing data

To test bias due to missing exposure data, we created nine additional exposure datasets for each county (Table 1), with varying levels of missingness. In each dataset, after generating outcomes based on complete data, we removed increasing percentages of county-hours to model missing data. In the scenario with the least missing data, 20% of counties were missing 20% of county-hours, and in the worst case, 80% of counties were missing 80% of county-hours.

**Table 1.** Simulated datasets to model missing data scenarios in an epidemiologic study of power outage exposure and hospitalizations

	Percentages of counties affected by missing data		
	20%	50%	80%
Percentages of county-hours missing in counties affected by missing data			
20%	Dataset 1: 20% of counties missing 20% of county-hours	Dataset 2: 20% of counties missing 50% of county-hours	Dataset 3: 20% of counties missing 80% of county-hours
50%	Dataset 4: 50% of counties missing 20% of county-hours	Dataset 5: 50% of counties missing 50% of county-hours	Dataset 6: 50% of counties missing 80% of county-hours
80%	Dataset 7: 20% of counties missing 80% of county-hours	Dataset 8: 50% of counties missing 80% of county-hours	Dataset 9: 80% of counties missing 80% of county-hours

To create missingness, we randomly removed county-hour observations from the original dataset according to each assumed missingness percentage. We treated missing observations as though they indicated no power outage exposure (0 customers without power) since this is the mean, median, and modal value of customers without power by county-hour in the POUS dataset. We applied our definition of power outage exposure to these nine datasets with missingness to create daily binary power outage exposure data based on a power outage duration of 8+ hours.

We then modeled the relationship between 8+ hour power outage exposure measured in the nine datasets with missing data and hospitalization counts generated based on an 8+ hour power outage exposure in the complete dataset without missingness. We estimated effects using both the difference-in-differences and case-crossover designs described above, and repeated simulations for three effect sizes, where the base hospitalization rate was 0.1%, and the hospitalization rate was increased to 0.101%, 0.105%, or 0.15%.

#### Methods summary

Currently available power outage datasets report the number of customers without power by hour and by county. When measuring power outage, researchers aim to capture power outage magnitude (how many people were without power) and power outage duration (for how long customers were without power). In the overview section, we suggested that researchers define daily power outage exposure by choosing a cut point (e.g., 10% of customers without power) and a duration (e.g., 8 hours) and considering a spatial unit exposed when greater than 10% of customers were without power for more than 8 hours on a given day.

Current data are also limited: many counties are missing up to 70% of county-hours and the health-relevant duration of power outage is unknown. We ran simulations where we tested how missing data could bias effect estimates from an epidemiologic study of power outage and a health outcome, and we tested how misidentifying the health-relevant duration of the power outage could bias effect estimates.

## Results

We ran a simulation representing an epidemiologic study of power outage and hospitalizations. We created 100 counties populated with electrical customers and simulated daily power outage exposure for these customers for 1 year. We simulated increased hospitalization rates resulting from these power outage exposures and estimated the effect of outage exposure on hospitalization rates. We repeated each simulation 100 times, meaning in total, we created 1000 simulated counties. The simulated counties contained an average of 360,000 electrical customers, who experienced a yearly average of 23 (SD: 32) 8+ hour power outages.

With these simulations, we aimed to quantify the bias introduced in this epidemiologic study when there was exposure misclassification in power outage exposure due to wrong assumptions about the health-relevant duration of power outage. We also aimed to quantify bias due to missing power outage data. We calculated bias using the absolute difference between the estimated and true simulated effects (β*−β, where β* is the estimated effect and β is the true simulated effect). We also assessed coverage of 95% confidence intervals in each of the simulations: the percentage of 95% confidence intervals including the true effect estimate.

We found evidence of bias in these simulations: results from both exposure misclassification and missing data scenarios were biased towards the null.

### Health-relevant duration

We observed substantial bias in the simulation scenarios representing incorrect assumptions about the health-relevant power outage duration. All effects reported here refer to the main simulation case with an effect size of 1% and a difference-in-differences design, unless otherwise stated.

**Figure 2.**
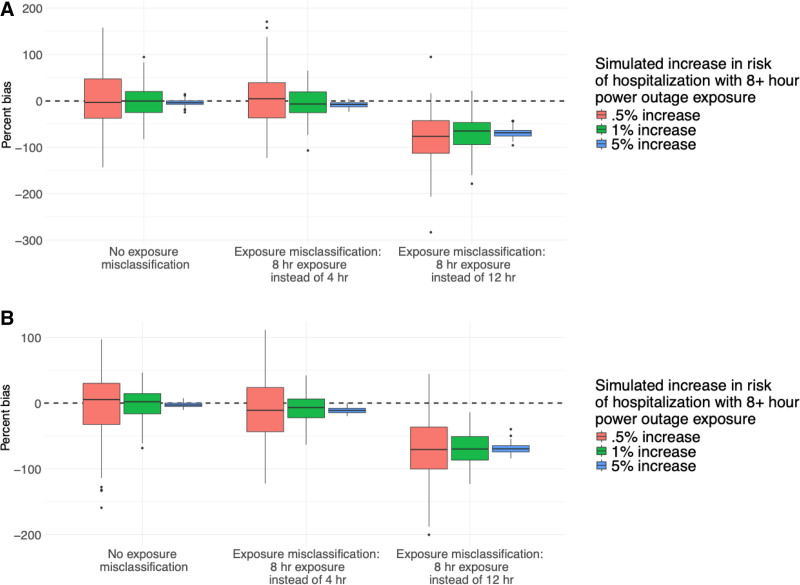
Results from simulations of the association between power outage exposure and hospitalizations in 100 counties for exposure misclassification scenarios representing wrong assumptions about the health-relevant duration of power outage, for a (A) difference-in-differences design, and (B) case-crossover design. Simulations were repeated 100 times, and boxplots show the distribution of the percent bias of effect estimates in each simulation scenario. There is a dashed line at 0.

When the assumed health-relevant power outage duration (8+ hours) was longer than the true health-relevant duration (4+ hours), the results were slightly biased towards the null (Figure 2). The mean bias of effect estimates was −6.7%, meaning that effect estimates were on average 93.3% of the true effect. The magnitude and direction of bias were similar across effect size and study designs.

However, when the assumed health-relevant duration of outage (8+ hours) was shorter than the true simulated duration (12+ hours), results were substantially biased toward the null. In this case, effect estimates returned by the simulation were on average 35.3% of the true simulated effect size (−64.7% bias in estimated coefficients). Again, the magnitude of bias was similar for all effect sizes and study designs. This was the largest bias observed across all exposure misclassification and missing data simulation scenarios. As effect size increased, the variability of bias decreased, across study designs (Figure 2).

**Figure 3.**
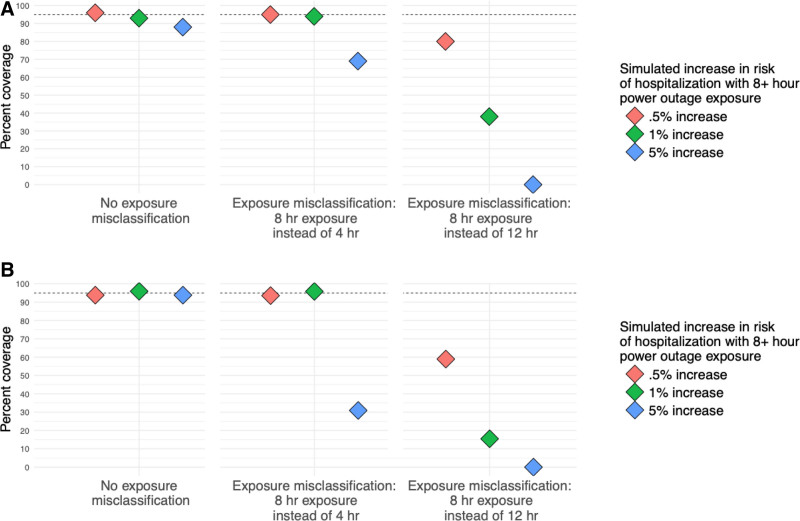
Results from simulations of the association between power outage exposure and hospitalizations in 100 counties with different percentages of exposure data missing using a (A) difference-in-differences design and (B) case-crossover design. Simulations were repeated 100 times, and plots show the percent coverage of 95% confidence intervals. There is a dashed line at 95%.

In scenarios modeling incorrect assumptions about the health-relevant duration, coverage of 95% confidence intervals varied widely by effect size and was different between the two exposure misclassification scenarios (Figure 3). When the assumed health-relevant duration of outage (8+ hours) was longer than the true simulated duration (4+ hours) coverage for models with a simulated effect size of 0.5% was 98%. For an effect size of 5%, coverage was 68%.

In the second health-relevant duration simulation, when 12+ hour power outages caused increased hospitalization risk, but the researcher assumed it was 8+ hour outages that caused health effects, coverage for models with simulated effect size 0.5% was 76%. However, for the effect size of 5%, coverage was 0%.

### Missing data

**Figure 4.**
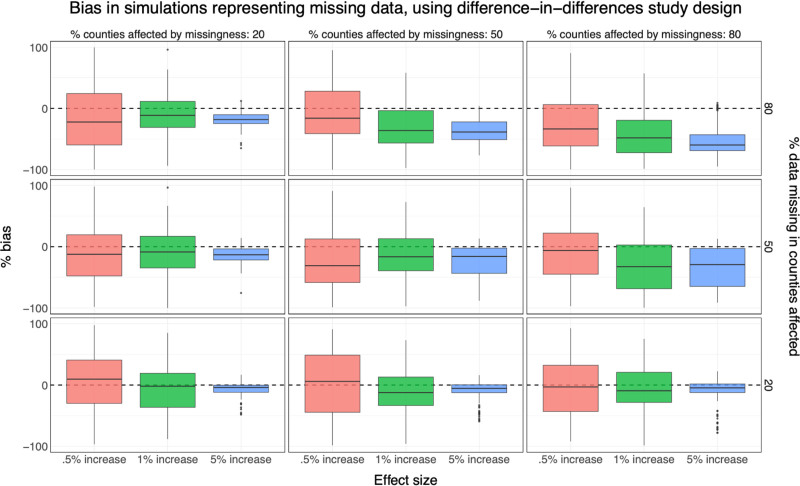
Results from simulations of the association between power outage exposure and hospitalizations in 100 counties with different percentages of exposure data missing for a difference-in-differences design. Simulations were repeated 100 times, and boxplots show the distribution of the percent bias of effect estimates in each simulation scenario. There is a dashed line at 0. Results correspond to Table 2.

In those simulations where missing data were introduced, as more data were missing, the estimated effect of outage on hospitalization was biased further toward the null (Figure 4). When 20% of counties were missing 80% of data, effect estimates returned from the simulation were 86.7% of the true simulated effect (i.e., −13.3% bias, SD: 37.2) for difference-in-differences study designs and effect size of 1% (Table 2). When 80% of data were missing from 50% of counties, effect estimates returned from the simulation were, on average, 67.0% of the true simulated effect (−33.0% bias, SD: 45.2), and when 80% of data were missing from 80% of counties, effect estimates were on average 45.6% of the true simulated effect (−54.4% bias, SD: 39.8). The magnitude of bias was not sensitive to study design or effect size. However, for smaller effect sizes, effect estimates were much more variable (Figure 4).

**Table 2.** Results from simulations of the association between power outage exposure and hospitalizations in 100 counties with different percentages of exposure data missing for a (A) difference-in-differences design, and (B) case-crossover design. Results correspond to column 3 of Figure 4

Effect size of power outage on hospitalization	0% of counties missing 0% of data	20% of counties missing 80% of data		50% of counties missing 80% of data		80% of counties missing 80% of data	
**A: Results from difference-in-differences study design simulations, select cases of missingness. Average percent bias (standard deviation of bias); coverage.**							
0.5%	−8.9% (83.6); 94.9%	−17.5% (74.6); 94.9%		−29.5% (85.4); 90.9%		−54.2% (81.1); 89.9%	
1%	0.4% (36.5); 93.9%	−13.3% (37.2); 96%		−33.0% (45.2); 81.8%		−54.4% (39.8); 63.6%	
5%	−1.3% (8.7); 88.9%	−18% (14.1); 40.4%		−35.5% (19.5); 19.2%		−50.5% (26.7); 18.2%	
**B: Results from case-crossover study design simulations, select cases of missingness. Average percent bias (standard deviation of bias); coverage.**							
0.5%	−9.2% (59.3); 95.8%		−22.1% (54.2); 92.7%		−34.0% (61.2); 87.8%		−57.6% (57.8); 80.9%
1%	0.3% (29.3); 95.7%		−16.4% (31.1); 90.5%		−31.7% (34.9); 73%		−51.8% (35.7); 49%
5%	−1.9% (5.7); 95.7%		−17.8% (13.1); 23.5%		−33.7% (18.7); 19%		−48.8% (25.6); 19.1%

**Figure 5.**
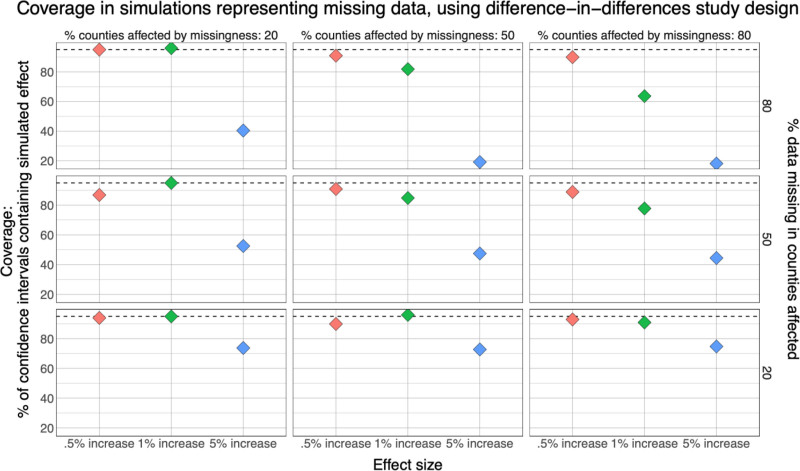
Results from simulations of the association between power outage exposure and hospitalizations in 100 counties with different percentages of exposure data missing using a difference-in-differences design. Simulations were repeated 100 times, and plots show the percent coverage of 95% confidence intervals. Results from case-crossover design are similar and are included in the Supplement; https://links.lww.com/EE/A357. There is a dashed line at 95%.

In these scenarios, coverage was high when effect sizes were small (Figure 5). When 80% of data were missing from 20% of counties, and the effect size was 0.5%, coverage was 94.9%. Coverage dropped substantially in all cases as effect size increased. When 80% of data were missing from 20% of counties and the effect size was 5%, although results were less biased than in higher missingness scenarios, coverage was 40.4%.

Coverage in missing data scenarios also decreased as the proportion of missing data increased, and coverage still depended on effect size. Coverage was close to 95% in all missing data scenarios when the effect size was 0.5% but decreased substantially as effect size increased and as more counties were affected by missing data, reaching a low of 18.2% when 80% of data were missing from 80% of counties in difference-in-differences models. Coverage was similar between study designs.

In summary, the largest bias was introduced from wrong assumptions about health-relevant power outage duration. There was also substantial bias as more missing data were introduced.

## Discussion

We developed a strategy to define and assess power outage exposure to support epidemiologic research. We calculated bias from misidentifying the health-relevant power outage duration and missing exposure data in simulations. Our exposure assignment strategy and simulation results will allow researchers to use available datasets to consistently assess power outage exposure while reducing potential bias in future epidemiologic studies.

To minimize bias, we recommend researchers avoid underestimating the health-relevant duration of power outage. If researchers are unsure of the health-relevant duration for their outcome, we recommend conducting sensitivity analyses varying the health-relevant duration or using a continuous measure of the daily number of hours without power to identify the health-relevant duration of outages. When assembling an analytic dataset of power outage exposure, we also recommend researchers investigate missingness in their power outage exposure data, and consider how much bias they can tolerate (Figure 4) while balancing generalizability.

We found evidence of bias in simulations where we modeled incorrect assumptions about the health-relevant power outage duration. Results were the most biased (mean bias: −64.7%, coverage: 46%) in those simulations representing a researcher underestimating the health-relevant duration of outage (when the assumed duration was 8+ hours but the true relevant duration was 12+ hours). Results were less biased (mean bias: −6.7%, coverage: 96%) when the assumed health-relevant duration of outages was longer (8+ hours) than the true health-relevant duration (4+ hours).

We also estimated substantial bias in simulations in which large percentages of exposure data were missing (50% missing from 50% of counties—80% missing from 80% of counties). The magnitude of bias did not depend on study design or effect size. However, as expected, coverage was low when the simulated effect size was larger and effect estimates were substantially biased, since these results were more precise than in simulations with smaller effect sizes.

All studies using the NYS power outage dataset have used similar (but not identical) definitions of power outage exposure to the one we proposed.^8,9,26^ Many of these studies have used a cut point-based definition under which spatial units are exposed to power outage when >*k*% of customers are without power, though details about the duration of power outage or the cut point have varied. According to our results, if any of these studies have misspecified the health-relevant duration of outage, their results could be biased toward the null.

To reduce bias due to missing data, researchers may exclude spatial units with high percentages of missing data. However, ignoring missing data or excluding spatial units with high percentages of missing data could result in selection bias or affect generalizability. We suggest that researchers carefully compare characteristics of included versus excluded spatial units to assess generalizability and interpret any results accordingly. Researchers could also conduct sensitivity analyses, comparing results from models including only spatial units with high person-coverage to results including spatial units with high and lower coverage.

Finally, we draw readers’ attention to the recently publicly available county-level 15-minute interval EAGLE-I dataset produced by the US Department of Energy and the Oak Ridge National Laboratory.^27^ This dataset is similar to the POUS dataset, since it reports customers without power by spatial unit, though at a finer temporal resolution. Researchers may want to apply the strategies suggested in this paper to assess exposure using this similarly structured dataset in addition to the available POUS and NYS datasets. Our results on the effects of assumptions on health-relevant duration and effects of missing data generalize to analyses conducted with the EAGLE-I dataset because of its similar structure to the POUS and NYS datasets.

## Limitations

First, in this study, we only assessed bias from data missing completely at random. In the POUS dataset, data may not be missing completely at random. Anecdotally, we have noticed some utility company websites are unavailable during large outages, suggesting that data could be missing more often from the POUS dataset during large outage events. We did not examine bias due to nonrandom data missingness mechanisms, but bias could be substantial in either direction. Future research should explore this possibility to improve recommendations for handling missing power outage data.

Second, studies using existing datasets measuring power outage exposure need to use aggregate, spatial unit measures of power outage to estimate effects, as there are no large-scale individual-level power outage datasets available. We did not assess how aggregating measurements from the individual to the spatial unit level could bias effect estimates. Future studies are needed to address this question, along with more detailed power outage exposure data.

Third, we opted to construct a binary power outage exposure variable. This construct captures the two dimensions of power outage exposure (magnitude and duration) and aligns with how many people think about power outages, making it more easily interpretable and policy-relevant. For some questions, a continuous measure of power outage exposure in a spatial unit (i.e., number of person-hours or hours above a cut point of power outage) may be more relevant. Specifically, when there is no *a priori* hypothesis about the health-relevant power outage duration, using a continuous exposure could help researchers identify the most health-relevant duration. Researchers will need to select the best definition of power outage exposure for their specific research question.

## Conclusion

To date, data availability has constrained research on power outages and health. In previous work, we developed a new national dataset of power outage exposure, the POUS dataset, which could expand the study of power outages and health outcomes. Because there is substantial missing data in the POUS dataset and no established method to assess power outage exposure in the literature, in this paper, we suggested a strategy to assess power outage exposure. Then, we used simulations to test how incorrect assumptions about health-relevant duration of power outage and missing data can bias the results of epidemiologic studies of power outage and health outcomes.

We found that there was substantial bias introduced in some cases, when the health-relevant duration of outage was assumed to be shorter than the true, and that bias increased as missing data increased. Our results show that while bias is likely, sensitivity analyses and careful choices of health-relevant duration can help researchers describe the range of plausible effect estimates in epidemiologic studies of power outage and health.

Despite the high percentage of missing data in the POUS dataset, the dataset is still high resolution, with hourly measurements in 2447 US counties over 3 years. Even after excluding counties missing >50% of exposure data, the dataset covers about 70% of the US population. We hope researchers can use our results to define and measure power outage exposure in future epidemiologic studies based on the POUS, EAGLE-I, and NYS datasets available while reducing potential bias.

## Conflicts of interest statement

The authors declare that they have no conflicts of interest with regard to the content of this report.

## Acknowledgments

*We thank Nina Flores, Alex Northrop, and Vivian Do for their continuing contributions to our lab’s work on power outage exposure.*

## Supplementary Material
