# Supplementary material for: Assessing bias in measuring power outage exposure with simulations
Source: Environ Epidemiol. 2025 Jun 11;9(4):e403. doi: 10.1097/EE9.0000000000000403 (PMC12160736; doi:10.1097/EE9.0000000000000403)
Supplement: Supplementary file 1 [file ee9-9-e403-s001.pdf]

Assessing potential sources of bias in measuring power outage exposure with simulations supplement

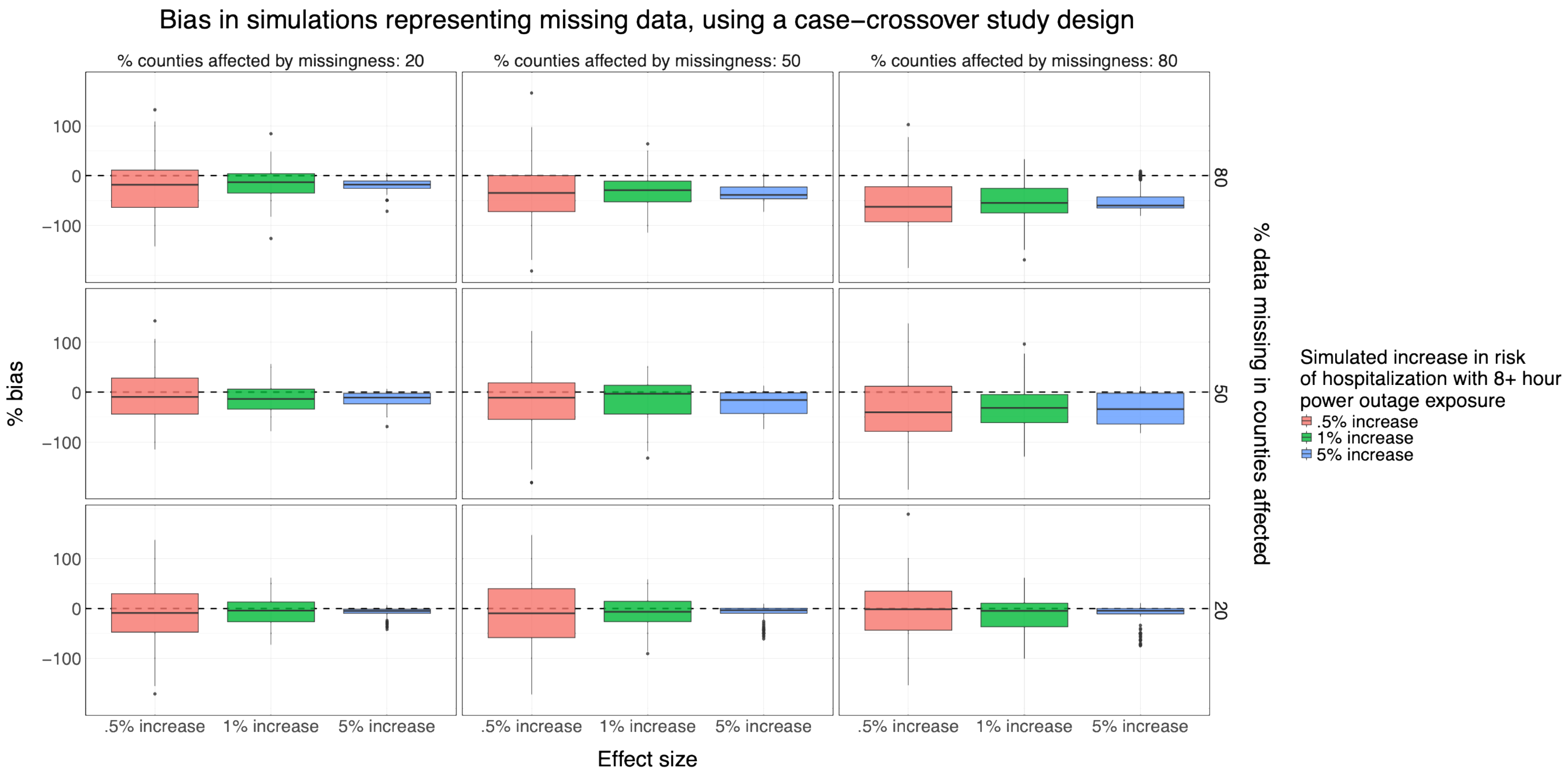

Supplement Figure 1: Results from simulations of the association between power outage exposure and hospitalizations in 100 counties with different percentages of exposure data missing, using a case-crossover design. Simulations were repeated 100 times, and boxplots show the distribution of the percent bias of effect estimates in each simulation scenario. There is a dashed line at 0.

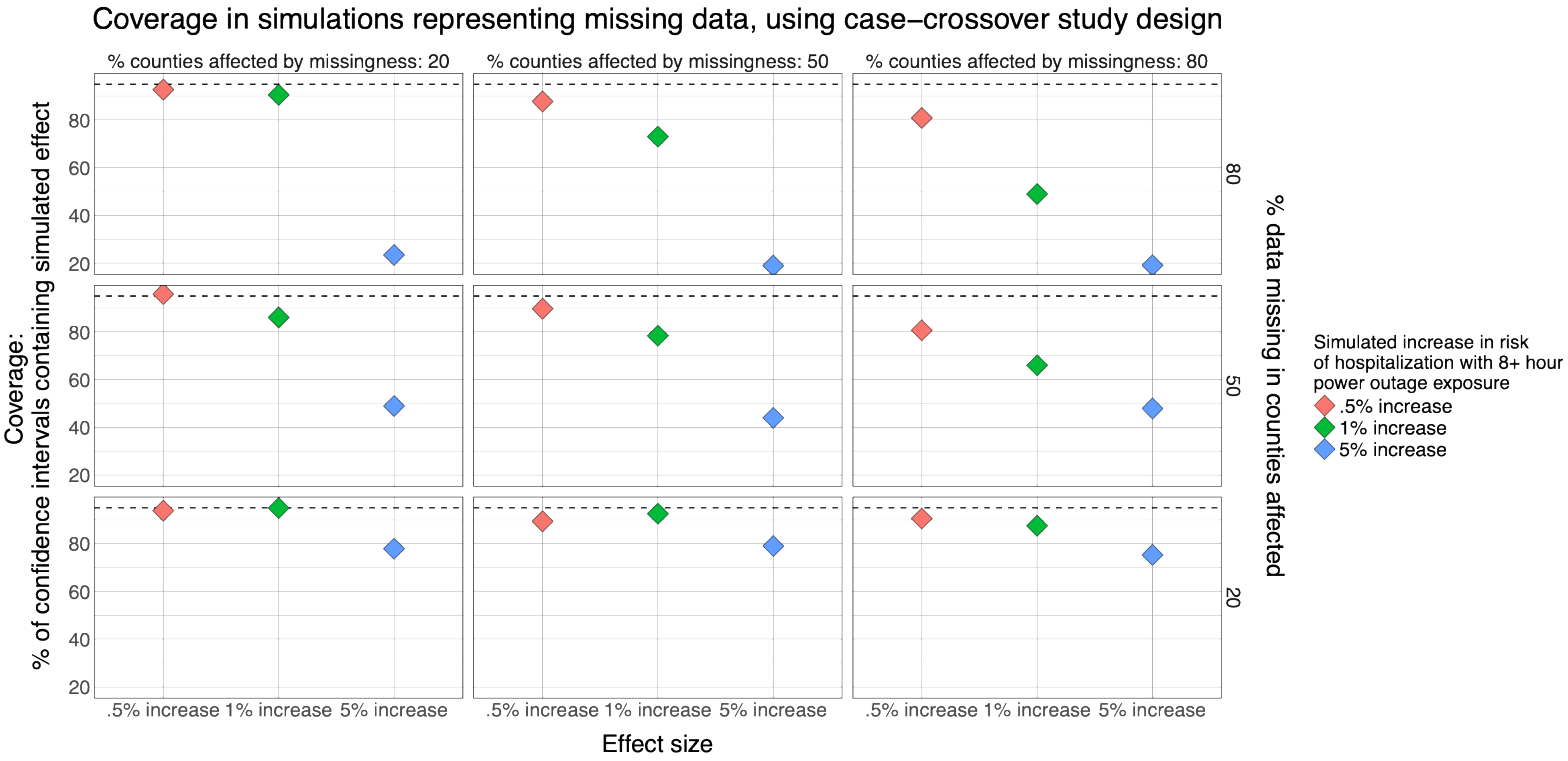

Supplement Figure 2: Results from simulations of the association between power outage exposure and hospitalizations in 100 counties with different percentages of exposure data missing using a case-crossover design. Simulations were repeated 100 times, and plots show the percent coverage of 95% confidence intervals. There is a dashed line at 95%

Supplement Table 1: Results from simulations of the association between power outage exposure and hospitalizations in 100 counties for exposure misclassification scenarios representing wrong assumptions about the health-relevant duration of power outage, for a (A) difference-in-differences design, and (B) case-crossover design. Simulations were repeated 100 times.

Table 1A: Results from difference-in-differences study design simulations. Average percent bias (standard deviation of bias); coverage.

Effect size	No exposure misclassification	Exposure misclassification: 8+ hour exposure instead of 4+ hour	Exposure misclassification: 8+ hour exposure instead of 12+ hour
0.5%	-11.5% (72.1); 93%	-10.6% (57.6); 98%	-63.8% (70.6); 76%
1%	2.3% (30.3); 95%	-6.7% (30.6); 96%	-64.7% (34.9); 46%
5%	-2.5% (5.8); 96%	-8.1% (7); 68%	-68.2% (9.2); 0%

Table 1B: Results from case-crossover study design simulations. Average percent bias (standard deviation of bias); coverage.

Effect size	No exposure misclassification	Exposure misclassification: 8+ hour exposure instead of 4+ hour	Exposure misclassification: 8+ hour exposure instead of 12 hr
0.5%	-3.4% (49.6); 93.8%	-9.4% (48.4); 93.5%	-72.1% (47.9); 58.9%
1%	-0.7% (22.1); 95.9%	-7.5% (21.4); 95.8%	-69% (24.1); 15.5%
5%	-2.6% (3.8); 93.8%	-11.1% (4.5); 30.9%	-69.1% (7.6); 0%
